# Uptake and Effects of Six Rare Earth Elements (REEs) on Selected Native and Crop Species Growing in Contaminated Soils

**DOI:** 10.1371/journal.pone.0129936

**Published:** 2015-06-15

**Authors:** David Carpenter, Céline Boutin, Jane E. Allison, Jessica L. Parsons, Deanna M. Ellis

**Affiliations:** 1 Science & Technology Branch, Environment Canada, Ottawa, Ontario, Canada; 2 Department of Biology, Ottawa—Carleton Institute of Biology, University of Ottawa, Ottawa, Ontario, Canada; 3 Department of Biology, Ottawa—Carleton Institute of Biology, Carleton University, Ottawa, Ontario, Canada; Banaras Hindu University, INDIA

## Abstract

Rare earth elements (REEs) have become increasingly important metals used in modern technology. Processes including mining, oil refining, discarding of obsolete equipment containing REEs, and the use of REE-containing phosphate fertilizers may increase the likelihood of environmental contamination. However, there is a scarcity of information on the toxicity and accumulation of these metals to terrestrial primary producers in contaminated soils. The objective of this work was to assess the phytotoxicity and uptake from contaminated soil of six REEs (chloride forms of praseodymium, neodymium, samarium, terbium, dysprosium, and erbium) on three native plants (*Asclepias syriaca* L., *Desmodium canadense* (L.) DC., *Panicum virgatum* L.) and two crop species (*Raphanus sativus* L., *Solanum lycopersicum* L.) in separate dose-response experiments under growth chamber conditions. Limited effects of REEs were found on seed germination and speed of germination. Effects on aboveground and belowground biomass were more pronounced, especially for the three native species, which were always more sensitive than the crop species tested. Inhibition concentrations (IC25 and IC50) causing 25 or 50% reductions in plant biomass respectively, were measured. For the native species, the majority of aboveground biomass IC25s (11 out of 18) fell within 100 to 300 mg REE/kg dry soil. In comparison to the native species, IC25s for the crops were always greater than 400 mg REE/kg, with the majority of results (seven out of 12) falling above 700 mg REE/kg. IC50s were often not detected for the crops. Root biomass of native species was also affected at lower doses than in crops. REE uptake by plants was higher in the belowground parts than in the above-ground plant tissues. Results also revealed that chloride may have contributed to the sensitivity of the native species, *Desmodium canadense*, one of the most sensitive species studied. Nevertheless, these results demonstrated that phytotoxicity may be a concern in contaminated areas.

## Introduction

Rare earth elements (hereafter referred to as REEs) are metals of the lanthanoid series in the periodic table. Though they are termed “rare”, REEs are in fact commonly found in soils worldwide [1]; the classification of “rare” solely refers to the lack of large deposits or ores that are characteristic of other elements such as silver and gold.

Once widely mined, China’s low production cost for REEs in the 1990s significantly reduced the prices of REEs globally and consequently many other mines stopped extracting these valuable elements [2]. As a result, China currently mines and produces approximately 95% or more of the world’s supply of REEs [3], and thus has a monopoly on these critical resources. However, in recent years China has reduced its production and export of REEs to protect its industry and to decrease the environmental impacts that may result from REE mining [4]. To supplement their supplies, other countries including Canada and the USA (a former producer of REEs) have begun the process of researching, developing or reopening REE mining facilities. Of particular interest in North America are sites at Thor Lake, Northwest Territories [5] and Strange Lake, Quebec [6] in Canada, and the pre-existing REE mine at Mountain Pass in California, USA [7].

REEs are mined primarily for their usefulness in modern technologies, with many applications for green-technology developments [8,9,10,11,12]. Their most important usages are as components of high strength magnets in electronic equipment, in wind turbines and electric vehicles, for precision guided weapons, and in computers, audio equipment and automobiles, amongst others. They are also used in low quantities as fluid cracking catalysts during oil refining, in the production of optical glass and as components in phosphors for energy efficient lighting [11].

The risks of REE pollution due to mining and processing as well as from the improper disposal of materials containing these compounds could potentially lead to elevated levels within the environment. In addition, the processing of REE rich monazite rocks for the production of phosphate fertilizers and the subsequent applications of these fertilizers could further elevate REE soil concentrations, especially in agricultural areas [13,14,15]. In Canada, these fertilizers are commonly used within agriculture in the prairies, with application levels in the late 1990s reaching 775 000 tonnes per year [16]. Sneller et al. [17] reported that approximately 85 tonnes of neodymium (Nd) were released into the environment from phosphate fertilizer production in the Netherlands in 1994. Slooff et al. [18] reported that industrial emissions in air and water due to fertilizer production in the Netherlands can contain over 500 mg/kg of REEs. Petroleum refining processes can release similar amounts of REEs into the environment. In the USA, an estimated 60–80 tonnes of REEs per day are released into the atmosphere by oil refineries [18].

In a study conducted by Li et al. [19], it was found that REE soil pollution due to tailings from an REE processing plant in China can travel up to approximately seven kilometers before soil concentrations stabilize to natural levels. Specifically, Nd and praseodymium (Pr) levels in relation to the source were found to be, respectively, 5726 and 1614 mg/kg at 0.4 km, 2266 and 650 mg/kg at 0.8 km, 1279 and 373 mg/kg at 1.3 km, and 310 and 85 mg/kg at 2.1 km [19]. Slooff et al. [18] also report that soils in polluted sites near industrial locations in the Netherlands contain high levels of REEs [800–900 mg/kg cerium (Ce), 500–700 mg/kg lanthanum (La), 400 mg/kg Nd and 100 mg/kg Pr], which are at least 100 times higher than background levels. Concentrations in mining areas in China reached upwards of 200 times that of baseline earth crust levels for most REEs, including Pr, Nd and samarium (Sm) [20]. For these reasons, toxicity monitoring will become crucial as REE mining activities commence in Canada and other countries.

Studies have indicated that REEs can be absorbed by plants due to the similar ionic radii that they share with calcium [21,22]. As a result, REEs may replace calcium molecules in a number of physiological processes involving proteins and enzymes, including root growth, photosynthesis, and flowering [15,21,23,24,25]. However, the mechanism of action of REEs in plants is still poorly understood [15]. Many studies have documented the presence of REEs in both the roots and shoots of a variety of different plant species; however, in these cases, the studies were conducted on plants growing in soils containing low, natural levels of the REEs [26,27,28,29]. Toxicological studies on the effects of REE soil contamination on plants are lacking, as the majority of research has been conducted under hydroponic growth conditions [21,30,31]. A wide range of reports from China, where REE fertilizers are regularly applied to crops, report stimulatory, positive effects of various REEs on different aspects of plant metabolism, growth and yield [22,32,33,34]. However, many of these positive effects are only observed at low doses of the REEs, with negative effects becoming apparent as dosages are increased [34]. Reported detrimental effects of elevated levels of REEs on plants include: decreased growth, root function and nutritional uptake [31,35]; reduced root elongation (erbium, Er [36]); decreased seed germination (La and mixed REE solution [37]); and chloroplast damage (terbium, Tb [38]).

The importance of studying plants in the environmental assessment of contaminants can often be overlooked in favor of other organisms. For instance, Li et al. [19] observed that the soil macrofauna diversity near a REE processing plant in China was decreased at high REE concentrations, but unfortunately plant biodiversity and toxicity was not assessed. Plants, however, can serve as strong indicators of environmental health since they are literally grounded and cannot escape the presence of contaminants. In addition, since they are the primary producers of many ecosystems they serve as a major entryway for many contaminants into the food chain. For instance, Cowgill [39] observed uptake of several REEs by water-lilies (*Nymphaea odorata* Aiton) and subsequently found these metals in aphids that fed on this species.

In a previous paper, two light rare earth elements (LREEs) elements (La and Ce) as well as yttrium (Y) were examined for their toxicity to crops and native plants [35]. In this companion study we seek to assess the uptake and phytotoxicity of three light and three heavy rare earth elements (HREEs), on the germination and biomass of five plant species (three wild, native Canadian species and two crops) grown in soils contaminated with increasing concentrations of REEs.

## Materials and Methods

### REE background information

Praseodymium (Pr), neodymium (Nd), and samarium (Sm) are members of the subgroup of LREEs whose atomic numbers span from 57 to 64. The LREEs are often found in bastnäsite and monazite minerals [8]. _60_Nd is one of the most abundant REEs. Natural crustal levels of Nd are approximately 40 mg/kg soil [40]; however, detected quantities vary greatly by both locality and base soil type [28,41,42]. Concentrations in natural soils range from 1.2 to 52.5 mg Nd/kg dry soil worldwide [43,44]. In contrast, the abundance of both _59_Pr and _62_Sm is lower at 8.20 mg/kg and 7.05 mg/kg, respectively [45]. Other documented background levels of Sm and Pr in soils range from 3.7 mg/kg and 4.5 mg/kg in Japan [46], respectively, to 20.93 mg/kg and 46.90 mg/kg in Germany [42], respectively. Terbium (Tb), dysprosium (Dy), and erbium (Er) are members of the HREE series (atomic numbers between 65 and 71). The HREEs are commonly found together within ionic adsorption clays and in the phosphate mineral xenotime [8,47]. _65_Tb with an average natural crustal abundance of 1.2 mg/kg soil is often less abundant than both _66_Dy and _68_Er that have natural abundances of 5.2 and 2.8 mg/kg respectively [45]. Documented natural soil concentrations of these elements from around the world range from 0.13 to 2.30 mg/kg for Tb, from 0.51 to 12.10 mg/kg for Dy, and from 0.35 to 6.20 mg/kg for Er [26,42].

### Soil preparation

An artificial soil was prepared following Environment Canada (EC) protocols [EC Formulation of Artificial Soil (SOP 15.09/1.3/S)] consisting of 10% peat (Premier Sphagnum Peat Moss, Rivière-du-Loup, Quebec, Canada), 20% pulverized Kaolin clay (Edgar Minerals Inc., Edgar, Florida, USA) and 70% silica sand (OptaMinerals, Waterdown, Ontario, Canada) by dry weight. New batches of soil were prepared for each experiment, except in the cases of Dy and Er where the experiments were run concurrently. To ensure soil homogenization, soil was prepared in small 2.7 kg batches that were thoroughly mixed using a commercial grade electrical mixer (Axis M-20, Axis Equipment, Montreal, Quebec, Canada). Five hundred milliliters of water was added to each batch of soil in order to attain an initial soil moisture content of approximately 20%. To adjust the pH of the soil to the desired pH range (approximately pH = 6), and to compensate for the acidity of the peat (pH varying from 3.10–4.17), calcium carbonate was added at a rate of 15.0–25.5 g per batch of soil. The resulting soil pH was 5.80 ± 0.03 to 5.85 ± 0.02. The cation exchange capacity (CEC) of this soil blend was previously found to be approximately 7.7 ± 0.3 meq/100 g (analyses performed by EXOVA laboratory, Ottawa, ON, Canada using the ammonium acetate extraction method [48]). Once mixed, all soils were placed into large plastic storage containers and were allowed to settle for at least two days prior to use.

### Plant species

Five plant species were tested in each experiment: three native, wild species with wide distribution ranges and of high ecological values as well as two crop species because crops are primarily used in toxicity testing. The native species consisted of common milkweed (*Asclepias syriaca* L.), showy ticktrefoil (*Desmodium canadense* (L.) DC.) and switchgrass (*Panicum virgatum* L.), and the two crops were radish (*Raphanus sativus* L.) and tomato (*Solanum lycopersicum* L.) (Table 1). *Desmodium canadense* and *P*. *virgatum* were selected based on their high coefficients of conservation [49] (Table 1), while *A*. *syriaca* was chosen based on its importance to native fauna (i.e. monarch butterfly). All native species were found to have seed germination rates of >70% in Petri-dish pre-trials. Due to a stratification requirement, seeds of *A*. *syriaca* were cold stratified in a 4°C refrigerator for approximately one to two months prior to the beginning of each experiment; no other seeds required stratification.

**Table 1:** Plant species tested in the rare earth element (REE) experiments.

Species	Common Name	Crop/Native	Family	CC Score^a^
***Asclepias syriaca* L.**	Common milkweed	Native	Asclepiadaceae	0
***Desmodium canadense* (L.) DC.**	Showy ticktrefoil	Native	Fabaceae	5
***Panicum virgatum* L.**	Switchgrass	Native	Poaceae	6
***Raphanus sativus* L. var. Sparkler**	Radish	Crop	Brassicaceae	N/A
***Solanum lycopersicum* L. var. Beefsteak**	Tomato	Crop	Solanaceae	N/A

^a^ CC Score—Coefficient of conservation. Available only for native (wild) species. Scores range between 0 and 10 [49]. Higher numbers indicate plants of higher conservation value as they have higher fidelities to specific sets of ecological/habitat variables.

### Experimental setup

For all experiments, the chloride hydrate forms of each REE (Table 2) were selected as the source compound of the REE due to their high solubility in water. All compounds were purchased from Sigma Aldrich Canada Co., Oakville, ON. For all REEs except Pr, seven nominal doses (plus controls) of the REE chloride hexahydrate were chosen following the same geometric progression of 1.9 in order to produce equivalent nominal doses for each compound (Table 2). In the case of Pr, since the true hydrate form was not clearly known (communication with Sigma Aldrich indicated that it was most likely a hexahydrate; however the heptahydrate is also common), the nominal doses for this compound were increased, but the same geometric progression of 1.9 was followed with the assumption that the source compound was of the hexahydrate form. The amount of (REE)Cl_3_·6H_2_O to add to each dose was determined based on the percentage of the specific REE in the given compound. For simplicity, all results are based on and reported in terms of mg REE/kg dry soil.

**Table 2:** Sources of rare earth elements (REEs) and range of doses used for all REE soil exposure experiments.

REE	REE Source Compound	Formula	CAS	Doses^a^ [mg (REE)Cl3·6H2O]	% REE^b^	Doses^a^ (mg REE/kg)
**Pr**	Praseodymium chloride hydrate	PrCl3·xH2O^c^	19423-77-9	76.2–3590.5	39.65	30.2–1423.6
**Nd**	Neodymium chloride hexahydrate	NdCl3·6H2O	13477-89-9	71.4–3360.4	40.20	28.7–1351.3
**Sm**	Samarium chloride hexahydrate	SmCl3·6H2O	13465-55-9	69.7–3279.0	41.20	28.7–1351.3
**Tb**	Terbium chloride hexahydrate	TbCl3·6H2O	13798-24-8	67.9–3175.1	42.56	28.7–1351.3
**Dy**	Dysprosium chloride hexahydrate	DyCl3·6H2O	15059-52-6	66.6–3134.9	43.11	28.7–1351.3
**Er**	Erbium chloride hexahydrate	ErCl3·6H2O	10025-75-9	65.6–3084.2	43.82	28.7–1351.3

^a^ In all cases, seven doses (+ controls) were used, with successive doses following a geometric progression of 1.9.

^b^ %REE refers to the ratio of the molar mass of the rare earth element to the molar mass of the REE source compound, and is given as a percentage.

^c^ The true hydrate form was not clearly known although it was most likely a hexahydrate.

To contaminate the soil, approximately 10.5 kg of soil, or the equivalent of 300 g of artificial soil per pot (pot dimensions: 10 cm x 10 cm x 9 cm), was added to a large plastic container. Measured amounts of the (REE)Cl_3_·6H_2_O (depending on the REE and the dose) were dissolved in one liter of water. This solution was then poured slowly into the container and mixed thoroughly into the soil. To further hydrate the soil, an additional two liters of water was added to each batch by rinsing the solution flask in order to also ensure that all metal residues reached the soil. Once thoroughly mixed, the soil was split between the 35 replicates in the dose treatment. This was repeated for all (REE)Cl_3_·6H_2_O treatments, starting with the smallest dose. Control treatments were prepared in a similar fashion, with the exception that only three liters of water was added to the soil in order to maintain hydration consistency with the (REE) Cl_3_·6H_2_O contaminated batches. For all doses (including the controls), one pot of excess soil was bagged and refrigerated for soil REE concentration analysis (pre-experiment samples). Once prepared, all soil pots were transferred to growth chambers (Conviron, model PGW36, Winnipeg, Manitoba, Canada) and were left to settle for 24 hours before seeds were planted.

Seven replicates per species were used for each dose as well as the controls giving a total of 35 pots per dose, for 280 pots in each experiment, and 1680 pots overall. For each species, five seeds were planted per pot/replicate, per dose for a total of 280 seeds per species per experiment. Artificial light within the growth chambers followed a 16 hour light:8 hour dark light-cycle, with an average photosynthetic active radiation of 314 ± 4 μmol photons/m^2^/s during the day. Temperature within the module averaged 26.4 ± 0.5°C during the day cycle and 15.1 ± 0.1°C during the night. In order to ensure uniformity of conditions and to prevent confounding environmental factors, all plant trays were rotated within the growth chamber on a weekly basis.

Analysis of soil samples for the presence of Pr, Nd, Sm, Tb, Dy, and Er was performed by Brooks Rand Labs (Seattle, Washington, USA) in order to validate the soil contamination method used in this experiment. Two soil samples from each dose (one pre-, one post-experiment) from each experiment were analyzed separately for their corresponding REE concentration using a modified USEPA Method [50]. The pre-experiment soil sample was collected from the soil during the initial contamination (as mentioned above). The post-experiment samples were obtained after the plants were harvested by homogenizing soil samples from all pots of a given dose and after removing any remaining plant debris.

To examine the possible effect of chloride (Cl) on plant growth as a result of using Cl forms of the REEs, an experiment was conducted on all plant species using calcium chloride (CaCl_2_). Five chloride doses (62.5, 125, 250, 500, and 1000 mg Cl/kg dry soil) and two control doses (one with CaCO_3_ added to balance the Ca level with the highest Cl dose, and one with no CaCO_3_ added) were used. All other methodologies followed those used for the REE experiments, though the resulting soil pH was slightly lower (approximately pH = 5.6).

### Germination and harvest

Seed germination was recorded daily for all species by checking each pot for the presence of emerging cotyledons. Approximately one week after the control pots for a given species had reached 70% seed germination, all pots for that species were thinned to one plant per pot. To prevent bias, a random number generator was used to determine which seedling was left in the pot. Due to the uneven germinating times and sizes of *D*. *canadense* seedlings within a given pot, the alternate approach of leaving the largest seedling was preferred for all REE experiments.

In a previous experiment [51], it was found that plants growing in the artificial soil (as per the EC protocol) and not supplied with nutrients failed to grow past the first true leaf stage, thus limiting the possibility of detecting any real toxicological effects on exposed plants in long-term studies. To supplement the low nutrient artificial soil, fertilizer (Plant-Prod 20-20-20 All Purpose Fertilizer) was added to all pots on day 16 for Sm, day 17 for Nd and Tb, and day 18 for Pr, generally after all species had been thinned to one seedling per pot (*D*. *canadense* in the Sm experiment was fertilized prior to thinning). Due to an error, all pots in the Er experiment were fertilized on day 0 (pre-thinning) while all those of Dy were fertilized on day 24 (post-thinning). Fertilizer was prepared by dissolving 7.8 g of the soluble fertilizer in 1 L of water to make a stock solution. From the stock solution, 8.30 mL (approximately 65 mg of fertilizer) was added to each pot by pipette. The amount of fertilizer was calculated based on the recommended fertilization rates for the crop species (10% of the recommended doses for radish, *R*. *sativus*, and tomato, *S*. *lycopersicum*, was applied).

All plants of a given species were harvested 28 days after their respective controls had attained 70% germination. All aboveground biomass was collected by cutting the plant at the base of the soil, rinsing with water to remove residual soil debris, and placing it in a paper bag. For *R*. *sativus*, the belowground bulb was also harvested, rinsed, and bagged. In addition, all plants from the controls, as well as the 2^nd^, 4^th^ and 6^th^ nominal doses were selected for root characterization for all species. Soil was separated from the roots delicately by hand in a water basin and the roots rinsed under running water to remove excess soil debris. The roots were bagged separately for biomass determination. All plant material (shoots and roots) was placed in a drying oven for at least two days at approximately 70°C prior to dry biomass measurements. After the biomass was recorded, all dried plant material was pooled by REE, type (shoot or root), dose, and species for REE uptake/accumulation analysis. Only the controls, 2^nd^, 4^th^, and 6^th^ doses were evaluated. For *R*. *sativus*, radish bulbs were included with the root portion of the corresponding samples for the root REE concentration analyses. All plant samples were analyzed by Brooks Rand Labs using ICP-MS, and were processed and stored according to their standard operating procedures and EPA methodology.

Due to an unknown event, several control plants of *A*. *syriaca* (n = 4), *D*. *canadense* (n = 4), *R*. *sativus* (n = 3), and *S*. *lycopersicum* (n = 3), all within the same control tray of the Nd experiment, became stunted and malformed near the mid-point of the experiment. Since we could not determine the reason behind this event, all affected replicates were eliminated from all biomass statistical analyses; seedling germination data was unaffected and was thus still included for these replicates. No plants of *P*. *virgatum* were affected, and therefore all seven replicates for this species were used in biomass related analyses. In addition, due to accidental damage incurred while thinning the pots to one seedling, one *P*. *virgatum* replicate from the 207.7 mg Pr/kg nominal dose was eliminated from all biomass (shoot and root) related analyses due to stunted growth. However, due to minimum biomass weight requirements for Pr plant concentration analyses, it was included in the sample that was analyzed by Brooks Rand Labs.

### Statistical analysis

Statistical analyses were performed in Systat 13 (Version No.13.00.05). All analyses were run separately for each REE evaluated.

#### Germination

Total percent germination was recorded for all pots over the course of each experiment. In addition, in order to determine if there were time delays in seed germination at different REE doses, speed of seed germination was calculated. This parameter is a more sensitive measure than total percent germination as it gives more weight to earlier germinating seeds and thus may detect subtle delays in germination even when all seeds germinate. Speed of germination was determined for all species based on the number of seedlings that germinated in each pot per day until the end of the experiment and was calculated using the formula:

$$\sum \left(\frac{N1}{1}+\frac{N2}{2}+\frac{N3}{3}+\ldots +\frac{Ni}{i}\right)$$

Where:

*N*1 = # of new seeds germinating on Day 1, *N*2 = # of new seeds germinating on Day 2, etc. and *Ni* = # of new seeds germinating on Day *i*

Speed of germination was determined individually for each pot. Seven values were thus obtained for each dose per species.

ANOVA, or the non-parametric Kruskal-Wallis test, was used to determine the effects of REE dose on the germination of each species. The Kruskal-Wallis test was only performed if the ANOVA model assumptions of normality of residuals (Shapiro-Wilk test) or homogeneity of variance (Levene’s test) could not be met, even after transformation of the data. In all cases where a significant effect (p < 0.05) was observed, post hoc comparisons (ANOVA: Dunnett’s one-sided; Kruskal-Wallis: Conover-Inman test) were used to determine which doses were significantly lower than the controls.

#### Biomass

Inhibition concentrations (IC25 and IC50) causing 25 or 50% reductions in plant aboveground biomass (including the radish bulb for *R*. *sativus*) respectively, as compared to the controls, were calculated using non-linear regression model analyses [52] when the model assumptions of homogeneity of variance (Levene’s test) and normality of residuals (Shapiro-Wilk test) were met. For the cases where the parametric model assumptions could not be met, even after data transformations, the nonparametric ICPIN program [53] was used to determine the IC values. Average measured concentrations in soil were used for calculating ICs (Table 3).

**Table 3:** Average measured concentrations of rare earth elements (mg REE/kg dry soil) in control and dosed soils as compared to the expected nominal doses.

		Average Measured Concentration (% Recovery)					
Dose	Dose^a^	Pr^a^	Nd	Sm	Tb	Dy	Er
Control	0.00	1.46	5.78	1.40	0.18	1.20	0.57
1	28.72	25.65(80)	26.1(71)	26.85(89)	23.25(80)	27.7(92)	25.8(88)
2	54.57	41.65(70)	65.1(109)	65.85(118)	37.0(67)	45.9(82)	40.95(74)
3	103.69	125.0(113)	101.0(92)	96.2(91)	79.05(76)	78.65(75)	78.75(75)
4	197.02	149.0(71)	188.5(93)	102.75(51)	152.5(77)	167.0(84)	146.5(74)
5	374.33	311.5(79)	319.0(84)	317.0(84)	326.0(87)	320.5(85)	328.0(87)
6	711.23	592.0(79)	819.0(114)	430.0(60)	497.0(70)	632.5(89)	738.0(104)
7	1351.34	1032.5(82)	1545.0(114)	1060.0(78)	1056.5(78)	1080.0(80)	1065.0(79)

Values for control soils represent the detected background REE levels in the artificial soil. Percent recovery for dosed soils, calculated as: (average [REE] measured at a given dose—average measured [REE] in control soils) / (nominal dose), are presented in parentheses.

^a^ Nominal doses for Pr were 0.00, 30.21, 57.40, 109.50, 207.68, 393.47, 747.66, and 1423.59; % recovery for Pr was based on these values.

No observed effect concentrations (NOEC) and lowest observed effect concentrations (LOEC) for aboveground biomass (including the bulb weight for *R*. *sativus*) were determined for all species using either ANOVAs when data met the assumptions for parametric analysis, or Kruskal-Wallis tests when the data failed to meet the model assumptions even after data transformations were tested. Either Dunnett’s one-way post hoc test (ANOVA) or the Conover-Inman test (Kruskal-Wallis) was used to determine which doses had significantly lower biomasses than the controls.

To determine differences in root growth amongst the doses of a given REE, ANOVA or Kruskal-Wallis test were performed on root dry biomass in accordance with the model assumptions. Comparisons between doses were made using Dunnett’s one-way post hoc test or Conover-Inman test. The radish bulb was included in this measure for *R*. *sativus*.

Effects of chloride on plant biomass were assessed using ANOVA and Tukey’s honestly-significant-difference post hoc test to detect significant differences between the Cl doses and the controls where applicable.

## Results

### REE soil concentrations

All REEs were detected in all soils analyzed by Brooks Rand Labs, including the control soils (Table 3). Recovery rates for the REE-spiked soils were consistently high (> 70% recovery), with lower than expected recovery rates only apparent for doses four and six in the Sm experiment and dose two in the Tb experiment. Since REEs were detected in the control soils, doses used for all statistical analyses also included these background levels.

### Seed germination

Percent seed germination was found to be unaffected by exposure to REEs (Kruskal-Wallis tests or ANOVA, p > 0.05 for all species-REE combinations, see S1 Table and S2 Table), with one exception. Slightly fewer *R*. *sativus* seeds germinated in the 1^st^ dose of the Tb experiment as compared to the controls (Kruskal-Wallis test, χ^2^ = 16.923, df = 7, p = 0.018). However, since no effects were observed at the higher doses, this was attributed to overall seed viability within that specific dose rather than to Tb toxicity.

Exposure to REEs in the soil did not have major negative effects on the speed of germination of most species (Table 4; S1 Table). As compared to the controls, only seeds of *R*. *sativus* in the Nd experiment and those of *S*. *lycopersicum* in the Er experiment were found to have reduced germination rates at the highest dose evaluated (1545 mg Nd/kg and 1065 mg Er/kg respectively; Table 4). In addition, though statistical negative effects were observed at lower doses for both *R*. *sativus* in Pr and Sm soils and *D*. *canadense* in Sm soils, the negative effect did not persist at the highest dose evaluated in each situation.

**Table 4:** Analyses (ANOVA or Kruskal-Wallis test) of the effects of rare earth element (REE) soil dosage on the speed of germination of five plant species.

REE	Species	Days to 70% germination of controls	df	F or χ^2^	p-value	Doses Sig., Negatively different from controls (mg REE/kg)
**Pr**	*A*. *syriaca*	6	7,48	0.98	0.455	None
	*D*. *canadense*	17	7,48	0.94	0.488	None
	*P*. *virgatum*	7	7,48	0.96	0.472	None
	*R*. *sativus*	3	7,48	4.66	<0.001	311.50; 592.00
	*S*. *lycopersicum*	7	7	11.76^a^	0.109	None
**Nd**	*A*. *syriaca*	6	7,48	1.45	0.210	None
	*D*. *canadense*	13	7,48	1.34	0.254	None
	*P*. *virgatum*	6	7,48	1.38	0.236	None
	*R*. *sativus*	3	7	16.39^a^	0.022	1545.00
	*S*. *lycopersicum*	10	7,48	2.18	0.053	None
**Sm**	*A*. *syriaca*	6	7,48	0.86	0.544	None
	*D*. *canadense*	16	7,48	2.55	0.026	102.75; 430.00
	*P*. *virgatum*	8	7	2.79^a^	0.904	None
	*R*. *sativus*	3	7,48	4.12	0.001	317.00
	*S*. *lycopersicum*	7	7,48	3.29	0.006	None^b^
**Tb**	*A*. *syriaca*	6	7	17.87^a^	0.013	None^b^
	*D*. *canadense*	14	7,48	1.02	0.428	None
	*P*. *virgatum*	7	7	11.78^a^	0.108	None
	*R*. *sativus*	3	7,48	0.77	0.612	None
	*S*. *lycopersicum*	8	7,48	1.60	0.159	None
**Dy**	*A*. *syriaca*	6	7	13.67^a^	0.057	None
	*D*. *canadense*	13	7,48	2.43	0.032	None^b^
	*P*. *virgatum*	8	7,48	0.70	0.671	None
	*R*. *sativus*	4	7	9.76^a^	0.203	None
	*S*. *lycopersicum*	7	7	10.16^a^	0.180	None
**Er**	*A*. *syriaca*	6	7,48	1.11	0.371	None
	*D*. *canadense*	17	7,48	1.58	0.164	None
	*P*. *virgatum*	10	7,48	1.00	0.442	None
	*R*. *sativus*	4	7	4.93^a^	0.668	None
	*S*. *lycopersicum*	7	7,48	3.45	0.005	1065.00

Post hoc tests [Dunnett's one-sided (ANOVA) or Conover-Inman (Kruskal-Wallis)] were performed in order to determine doses that negatively differed from the controls. Additional information on calculated speed of germination is available in S1 Table.

^a^ Indicates χ^2^ value for Kruskal-Wallis analyses.

^b^ Though a statistical effect is present (p < 0.05), no doses were found to be significantly, negatively different from the controls.

### Aboveground biomass—Inhibition concentrations

Effects on shoot biomass, as determined through IC analyses, varied between REEs and between species within a given REE (Table 5). For all REEs, the three native species, *A*. *syriaca*, *D*. *canadense*, and *P*. *virgatum*, were always more sensitive than the two crops, *R*. *sativus* and *S*. *lycopersicum*. For the native species, the majority of IC25s (11 out of 18) fell within 100 to 300 mg REE/kg dry soil (all doses mentioned hereafter are in relation to dry soil concentration). In four cases (*D*. *canadense* in Sm, *A*. *syriaca* and *D*. *canadense* in Tb, and *P*. *virgatum* in Er) IC25s were found to be less than 100 mg REE/kg. Larger effects on biomass, as indicated by the IC50s, generally occurred at doses above 400 mg REE/kg, though IC50 effects were seen at smaller doses for *D*. *canadense* in both Sm and Tb soils. Overall, *D*. *canadense* was the most sensitive species.

**Table 5:** Summary of inhibition concentration (IC) values, calculated as the dosage (mg REE/kg dry soil) resulting in either a 25% (IC25) or 50% (IC50) decrease in the dry biomass of exposed plants as compared to the controls, for all plant species grown in rare earth element (REE) contaminated soils.

REE	Species	Model [Transformation]	IC25	IC25 95% CI	IC50	IC50 95% CI	R^2^
**Pr**	*A*. *syriaca*	Logistic[None]	260.82	138.96–488.78	523.81	340.98–804.38	0.464
	*D*. *canadense*	ICPIN	146.67	136.50–311.68	520.43	410.72–665.35	N/A
	*P*. *virgatum*	Gompertz[None]	199.91	73.64–541.00	547.28	307.32–971.75	0.401
	*R*. *sativus*	ICPIN	930.11	NC^b^	No Effect	N/A	N/A
	*S*. *lycopersicum*	ICPIN	651.23	457.14–752.18	No Effect	N/A	N/A
**Nd**	*A*. *syriaca*	Logistic[None]	216.27	89.99–517.80	600.17	330.89–1085.43	0.516
	*D*. *canadense*	ICPIN	232.45	24.96–386.08	644.65	274.36–875.39	N/A
	*P*. *virgatum*	ICPIN	260.10	60.50–333.73	550.19	408.54–913.53	N/A
	*R*. *sativus*	ICPIN	541.88	370.36–911.43	1081.68	650.03–1411.86	N/A
	*S*. *lycopersicum*	Gompertz[None]	1024.65	834.60–1260.82	1481.52	1320.30–1666.25	0.672
**Sm**	*A*. *syriaca*	Logistic [None]	254.86	163.44–396.19	455.04	342.56–605.74	0.607
	*D*. *canadense*	Gompertz [None]	19.56	7.73–47.42	89.99	47.42–170.00	0.688
	*P*. *virgatum*	Gompertz [None]	440.57	212.80–908.91	775.25	487.65–1234.95	0.266
	*R*. *sativus*	Gompertz [None]	517.80	35.39–7378.04	No Effect	N/A	0.286
	*S*. *lycopersicum*	ICPIN	457.35	226.30–641.84	No Effect	N/A	N/A
**Tb**	*A*. *syriaca*	Gompertz[SQRT]	66.61	32.57–135.14	403.58	250.19–649.13	0.730
	*D*. *canadense*	ICPIN	50.78	12.00–165.57	225.99	156.62–277.87	N/A
	*P*. *virgatum*	Logistic[None]	398.02	141.23–1118.44	No Effect	N/A	0.234
	*R*. *sativus*	Logistic[None]	No Effect^a^	N/A^c^	No Effect	N/A	0.064
	*S*. *lycopersicum*	Gompertz[None]	575.77	367.13–904.73	No Effect	N/A	0.454
**Dy**	*A*. *syriaca*	Gompertz[None]	272.53	34.65–2093.11	No Effect	N/A	0.194
	*D*. *canadense*	Gompertz[None]	212.30	72.28–618.44	757.58	407.32–1408.29	0.425
	*P*. *virgatum*	ICPIN	741.16	44.69–967.72	No Effect	N/A	N/A
	*R*. *sativus*	None	No Effect	N/A	No Effect	N/A	N/A
	*S*. *lycopersicum*	ICPIN	No Effect	N/A	No Effect	N/A	N/A
**Er**	*A*. *syriaca*	Logistic[None]	142.22	71.61–282.14	401.72	253.10–637.26	0.646
	*D*. *canadense*	Gompertz[None]	125.77	52.21–301.69	439.55	259.62–743.73	0.607
	*P*. *virgatum*	ICPIN	75.75	19.41–389.66	778.11	278.51–924.55	N/A
	*R*. *sativus*	ICPIN	No Effect	N/A	No Effect	N/A	N/A
	*S*. *lycopersicum*	Gompertz[None]	No Effect	N/A	No Effect	N/A	0.226

Dry biomass includes all aboveground plant tissues (shoots), as well as the radish bulb for *R*. *sativus*. IC values were calculated through parametric, non-linear regression models [52] or through the non-parametric ICPIN approach [53]. The 95% confidence intervals (CI) were determined through the Wald method for parametric data or through boot-straps for ICPIN data.

^a^ No effect. Either the IC value could not be determined, or the predicted value exceeded the range of doses evaluated in the experiment.

^b^ NC = Not calculable. ICPIN could not accurately predict CIs due to too few doses exhibiting IC25 effects.

^c^ N/A = Not available.

In comparison to the native species, IC25s for the crops were always greater than 400 mg REE/kg, with the majority of results (seven out of 12) falling above 700 mg REE/kg. IC50s could only be calculated for the crops in the Nd experiment, likely due to the higher concentration of that specific REE in the final dose as compared to the other REE experiments. In all other cases, predicted 50% effects on crop biomass either fell outside of, or were not apparent within, the range of doses evaluated.

**Fig 1:**
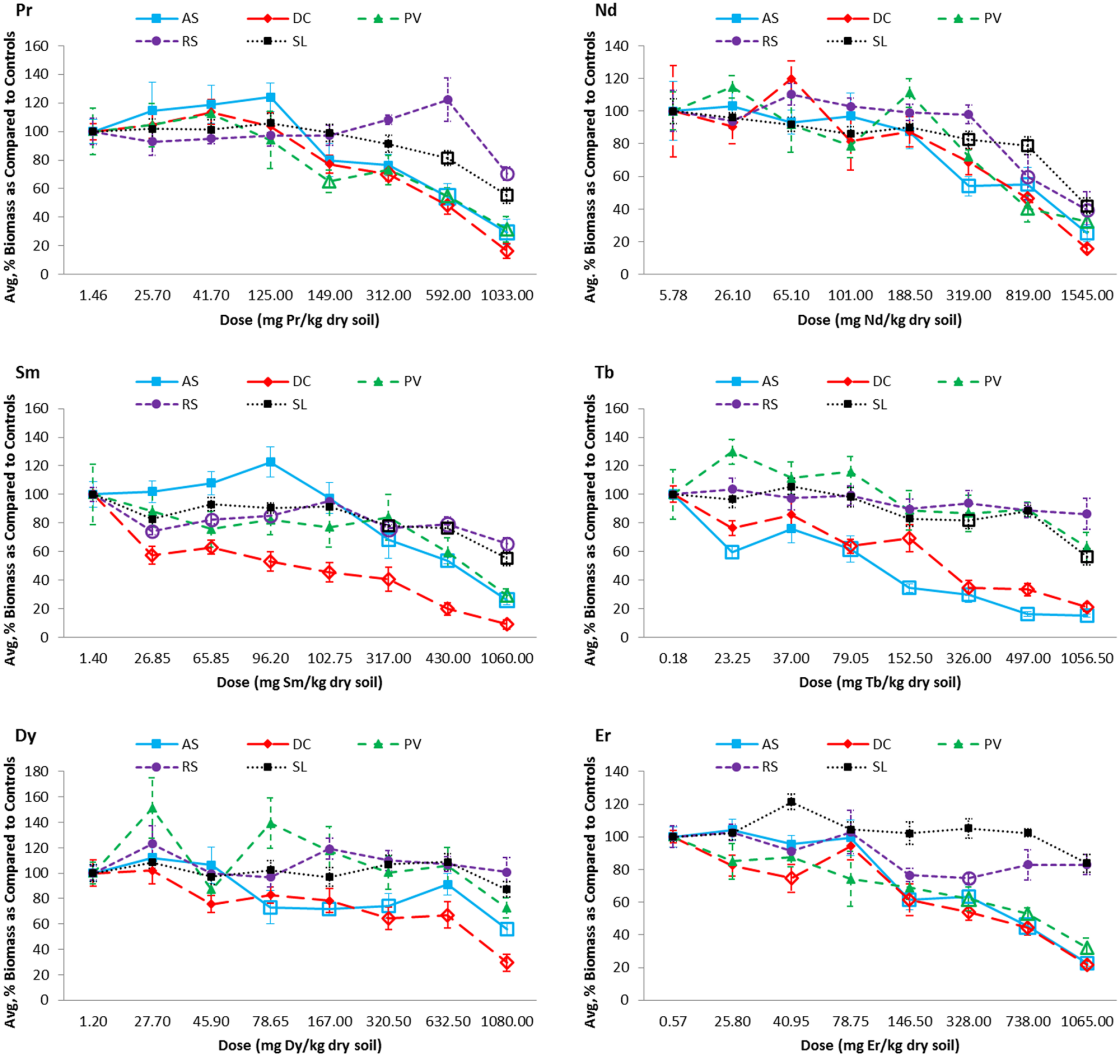
Dose-response curves for the aboveground, shoot (leaves and stems) biomass of all five tested plant species grown in rare earth element (REE) contaminated soils. Biomass is presented as the average percent biomass as compared to the controls, such that the controls always have a mean of 100%. Doses that were found to be significantly, negatively different from the controls based on statistical analyses using ANOVA (Dunnett's one-way post hoc comparison) or the Kruskal-Wallis test (Conover-Inman post hoc comparisons) are indicated with open bullets. Error bars represent standard error. AS = *Asclepias syriaca*, DC = *Desmodium canadense*, PV = *Panicum virgatum*, RS = *Raphanus sativus* and SL = *Solanum lycopersicum*.

The REE dose at which significant reductions in biomass (as compared to the controls) were first detected vary both by species within a given REE treatment and for a given species across all REEs (Fig 1; S3 Table). Generally, negative effects of Pr and Nd on plant biomass first appear at higher doses (149.00 and 319.00 mg REE/kg respectively) than for Sm (*D*. *canadense* and *R*. *sativus* at 26.85 mg Sm/kg), Tb (*A*. *syriaca* at 23.25 mg Tb/kg), Dy (*A*. *syriaca* at 78.65 mg Dy/kg), and Er (*D*. *canadense* at 40.95 mg Er/kg). However, though effects were observed at lower doses for Tb, Dy, and Er, not all species experienced significant reductions in biomasses for these REEs as they did for Pr, Nd, and Sm. By species, REE doses causing a statistically significant biomass reduction spanned from 23.25 (Tb) to 592.00 (Pr) mg REE/kg for *A*. *syriaca*; from 26.85 (Sm) to 819.00 (Nd) for *D*. *canadense*; from 149.00 (Pr) to 1060.00 (Sm) or no effect (Tb and Dy) for *P*. *virgatum*; from 26.85 (Sm) to 1032.50 (Pr) or no effect (Tb and Dy) for *R*. *sativus*; and from 317.00 (Sm) to 592.00 (Pr) or no effect (Dy and Er) for *S*. *lycopersicum*. As compared to their corresponding IC25 values (Table 5), the lowest dose at which ANOVA or Kruskal-Wallis analyses predict as being statistically lower than its corresponding controls (Fig 1, S3 Table) is generally greater than its IC25 value. Interestingly, for *P*. *virgatum* grown in Tb and Dy soils, though IC25 values were determined, no statistical differences between doses were detected.

### Belowground (root) biomass

**Fig 2:**
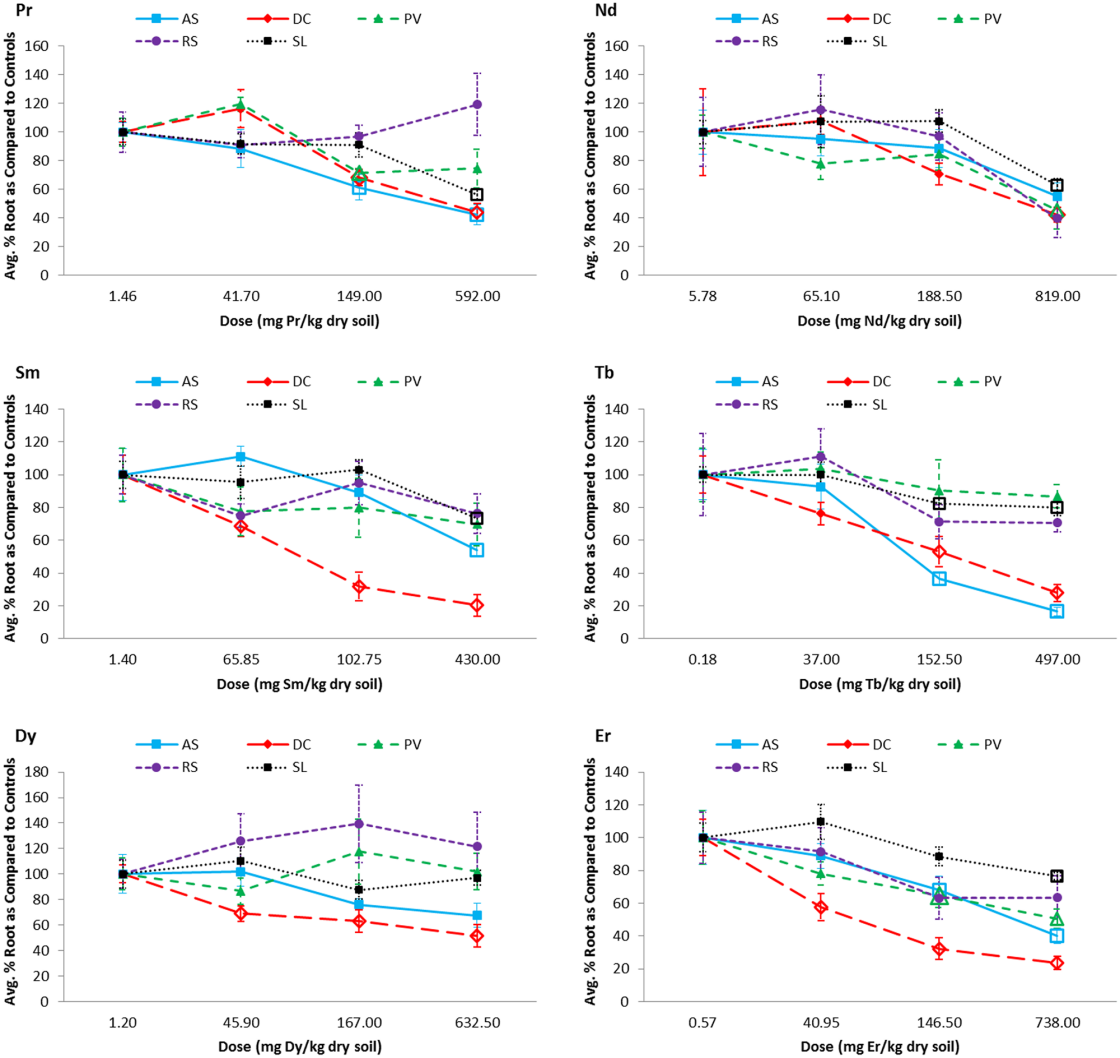
Dose-response curves for the belowground, root dry biomass (and including the radish bulb for *Raphanus sativus*) of all five tested plant species grown in rare earth element (REE) contaminated soils. Only the controls, 2^nd^, 4^th^, and 6^th^ REE doses were evaluated. Biomass is presented as the average percent biomass as compared to the controls, such that the controls always have a mean of 100%. Doses that were found to be significantly, negatively different from the controls based on statistical analyses using ANOVA (Dunnett's one-way post hoc comparison) or the Kruskal-Wallis test (Conover-Inman post hoc comparisons) are indicated with open bullets. Error bars represent standard error. AS = *Asclepias syriaca*, DC = *Desmodium canadense*, PV = *Panicum virgatum*, RS = *Raphanus sativus* and SL = *Solanum lycopersicum*.

Root biomass of the native species was generally affected at lower REE doses than the crops (Fig 2; S4 Table). *Desmodium canadense* was often the most sensitive species, being the only species with significant biomass reductions (as compared to the controls) detected at doses less than 100 mg REE/kg (Sm, Dy, and Er). In addition, *D*. *canadense* was the only species for which all REEs had a significant effect on root biomass in at least one dose, though the dosage at which the effects were noticeable varied considerably between the REEs (ranging from 40.95 mg/kg for Er to 819.00 mg/kg for Nd; Fig 2; S4 Table). Conversely, root biomass of *R*. *sativus* (including the radish bulb) was unaffected by all REEs within the range of doses for which it was evaluated. For the remaining species, REE concentrations causing significant effects on root biomass ranged from 146.50 (Er) to 430.00 mg REE/kg (Sm) for *A*. *syriaca*, with no effects observed in Nd or Dy; from 146.50 (Er) to 819.00 mg REE/kg (Nd) for *P*. *virgatum*, with no effects observed in Sm, Tb, or Dy; and from 152.50 (Tb) to 819.00 mg REE/kg (Nd) for *S*. *lycopersicum*, with no effects observed in Dy.

### Chloride experiment

Results revealed that there were no consistent significant differences among doses of calcium chloride and control with calcium carbonate in four species (although differences were observed with controls with no additional calcium added). However, significant differences were observed for *D*. *canadense* at doses from 125 mg/kg CaCl_2_ to 1000 mg/kg CaCl_2_ (S1 Fig).

### REE plant accumulation

REEs were detected in all root and shoot samples analyzed, including in the controls (Table 6). Measured concentrations in the shoots and roots always increased as the REE concentration increased, with the only exception occurring for *R*. *sativus* roots in Sm soils (measured concentrations dropped from 8.060 to 1.750 mg Sm/kg dry biomass between the 2^nd^ and 4^th^ doses). Measured concentrations were always found to be greater in the roots than in the shoots for any given dose, with three exceptions: *A*. *syriaca* grown in the Tb control soils (0.18 mg Tb/kg soil), *P*. *virgatum* in the Dy control soils (1.20 mg Dy/kg soil), and *R*. *sativus* in the 102.80 mg Sm/kg dosed Sm soil. Overall, no species showed any distinct trend in REE root accumulation (i.e. lower vs. higher accumulation rates) at any given REE soil concentration. However, at any given soil dosage for a given REE it was observed that the crop *R*. *sativus* often had the highest concentration in its shoots (13 out of 24 cases), whereas the grass *P*. *virgatum* often had the lowest detected shoot concentration (19 out of 24 cases).

**Table 6:** Rare earth elements (REE) soil concentration (mg REE/kg dry soil) and measured concentrations (mg REE/kg dry biomass) in the roots and shoots (aboveground biomass) of the five plant species grown in REE contaminated artificial soil.

REE	REE soil concentration	*A*. *syriaca*		*D*. *canadense*		*P*. *virgatum*		*R*. *sativus*		*S*. *lycopersicum*	
		Root	Shoot	Root	Shoot	Root	Shoot	Root	Shoot	Root	Shoot
**Pr**	1.46	0.120	0.050	0.140	0.050	0.280	0.100	0.130	0.040	0.100	0.060
	41.70	9.630	0.420	10.800	0.470	9.550	0.330	11.200	0.900	4.870	0.740
	149.00	37.000	2.340	30.900	2.610	30.200	2.050	24.200	4.780	19.900	3.560
	592.00	198.000	7.020	126.000	11.600	96.200	3.660	33.600	11.000	82.800	14.800
**Nd**	5.78	0.670	0.170	1.430	0.070	2.130	0.290	0.510	0.130	0.420	0.090
	65.10	10.300	0.430	11.300	0.470	20.100	0.370	13.600	1.290	17.400	0.470
	188.50	41.600	3.010	48.200	2.110	53.900	1.790	38.300	4.210	60.500	3.020
	819.00	176.000	7.180	223.000	8.450	287.000	6.690	170.000	25.500	135.000	12.800
**Sm**	1.40	0.300	0.080	0.060	0.020	0.160	0.030	0.140	0.010	0.260	0.040
	65.85	2.660	0.340	2.830	0.380	2.160	0.140	8.060	0.770	3.520	0.440
	102.75	10.300	0.900	15.200	1.550	6.980	0.470	1.750	3.230	19.900	1.050
	430.00	49.300	3.340	67.300	5.430	37.400	1.780	31.800	10.900	45.500	5.290
**Tb**	0.18	0.009	0.020	0.010	0.006	0.030	0.030	0.040	0.020	0.060	0.050
	37.00	1.310	0.340	1.810	0.310	2.670	0.130	6.140	0.450	3.180	0.360
	152.50	4.150	1.040	9.150	1.590	12.900	0.160	26.800	1.730	11.900	1.710
	497.00	73.500	5.330	49.000	10.300	28.900	1.330	65.500	7.580	50.300	7.660
**Dy**	1.20	0.080	0.020	0.170	0.020	0.100	0.130	0.120	0.030	0.140	0.020
	45.90	1.870	0.490	5.610	0.990	2.210	0.450	2.410	0.880	4.150	0.590
	167.00	7.710	1.520	18.300	2.280	5.440	0.980	15.000	2.750	14.600	2.150
	632.50	36.900	5.180	80.000	10.100	21.800	2.850	30.300	11.500	50.000	10.000
**Er**	0.57	0.050	0.020	0.070	0.010	0.080	0.009	0.020	0.010	0.070	0.030
	40.95	1.150	0.880	4.410	2.730	2.150	0.240	3.510	1.360	4.480	1.070
	146.50	5.650	3.160	12.000	3.870	11.200	0.650	13.400	3.380	15.500	3.030
	738.00	19.800	5.700	44.000	7.150	10.900	2.130	42.900	8.980	47.300	10.900

Numbers represent the results of chemical analyses of pooled plant samples corresponding to the plant species and REE dose combination.

REE Root:soil and shoot:soil ratios were fairly consistent within a given species for each REE, with no obvious increases or decreases with increasing soil concentrations (S5 Table). These ratios were also fairly consistent across REEs. The root:soil ratios (or transfer factor) ranges were: 0.07–0.33 (Pr), 0.07–0.35 (Nd), 0.03–0.19 (Sm), 0.03–0.18 (Tb), 0.03–0.14 (Dy), and 0.01–0.14 (Er) and the shoot:soil ratios: 0.008–0.068 (Pr), 0.006–0.050 (Nd), 0.002–0.057 (Sm), 0.001–0.021 (Tb—though much higher ratios were observed in the control dose), 0.005–0.108 (Dy), and 0.003–0.067 (Er).

## Discussion

Due to the increasing worldwide demands for REEs in modern technology, global REE mining and extraction activities have been on a steady rise. Though they have become widely used, very little is currently known about the potential environmental impacts and toxicities of these elements to native plants growing in contaminated soils, thus warranting further environmental monitoring.

### Effects on germination

Germination rates of all species were generally unaffected by REE soil concentrations. Some negative effects on speed of germination were identified for both *R*. *sativus* in Nd soils and *S*. *lycopersicum* in Er soils, but only at the highest dosage evaluated. Likewise, while effects were observed at intermediate doses for both *R*. *sativus* in Pr soils and *D*. *canadense* in Sm soils, the effect was not apparent at the highest dose. Due in part to these inconsistencies, it is not possible to rule out that these observed negative effects were due to variability in seed viability as opposed to REE toxicity. The results obtained in this experiment are similar to those found in a previous experiment with lanthanum (La), where no effects on germination were observed; however, the range of doses tested in the La study was significantly lower [35]. In contrast, two other REEs, yttrium and cerium, were observed to negatively affect the germination rates of the same species evaluated in the present study [35].

There is a paucity of studies on the effects of REEs on germination and early growth. Hu et al. [22,30] conducted studies in nutrient solutions rather than in soil and demonstrated both growth stimulation and inhibition. In contrast, D’Aquino et al. [37] observed reduced germination of *Triticum durum* Desf. seeds placed on germination paper after soaking in a La^3+^ or a combined REE solution, with the observed negative effects varying by both seed soaking times (2–8 hours) and by concentration (0.01–10 mM). In the current experiment, when seeds were grown directly in REE contaminated soils, no significant stimulation in germination was observed for any of the species and REEs studied. Though the results indicate a potential slight toxic effect of some REEs on the seeds of certain species, the high soil dosage required to elicit these effects would be more representative of levels found at contaminated sites near mining or processing facilities [19,20], as opposed to sites with more subtle inputs (e.g. fertilized lands, landfill leaching).

### Effects on aboveground and belowground biomass

High soil concentrations of the six REEs were needed to reduce the aboveground biomass of all plant species (as determined through IC25 and IC50 analyses). Native species were found to be significantly more sensitive to REEs (IC25s from 146.67 to 741.16 mg/kg dry soil) than crops (IC25s from 651.23 mg/kg dry soil to no effect). The same trend was observed for the IC50s. Likewise, effects on root biomass were more noticeable in native species than in the crops. Nevertheless, it is believed that significant quantities of REEs would have to be released into the environment to attain these potentially toxic levels. For instance, according to the information provided in Slooff et al. [18], the detected IC25 and IC50 values in this study are generally greater than the Nd and Pr soil concentrations reported at contaminated sites in the Netherlands (i.e. 400 mg Nd/kg soil and 100 mg Pr/kg soil), with the exceptions of the results for the three native species grown in Nd-spiked soils. In contrast to the HREEs (Tb, Dy and Er), levels of the LREEs (Pr, Nd and Sm) in contaminated sites in China exceeded both the EC25s and EC50s of most of the plant species investigated in this study [20]. Information on soil pollution levels of Sm, Tb, Dy and Er is scarce. Low to medium background concentrations of these REEs have been reported in natural soils, with high degrees of variability (0.51–20.93 mg Sm/kg soil, 0.13–2.3 mg Tb/kg soil, 0.51–12.1 mg Dy/kg soil and 0.16–6.2 mg Er/kg soil; see summaries in [54,55]). If a contamination factor of 100 times the background level is considered (as for Nd and Pr in the Netherlands [18]), it is likely that some plant species will be affected by these REEs; however, more site-specific baseline data from Canada and elsewhere around the world is necessary in order to form conclusions about their potential environmental impacts. In addition, further research on native species is required to properly measure hazards on other plant groups, such as bryophytes, pteridophytes, and woody plants.

### Chloride effect

In most cases, *D*. *canadense* was the most sensitive species tested (four cases for both the IC25 and IC50). However, the lower values observed for this species may have partially been the result of the significant sensitivity of this species to the chloride molecule. Damage to one-year old avocado (*Persea americana* Mill.) and citrus (*Citrus* L. spp) plants in response to increased concentrations of chloride in irrigation water has been demonstrated in previous studies ([56] and references therein). In another experiment, it was shown that accumulation of chloride caused a reduction in gas exchange within leaves, as well as interacted with sodium to reduce other physiological processes in *Citrus sinensis* (L.) Osbeck cv. Hamlin seedlings [57]. The adverse effect of chloride associated with REEs had not been previously investigated. Our findings suggest that other experiments performed under hydroponic or soil conditions and which showed negative effects on plants [58,59,60,61,62] may be partly related to the chloride component of the tested compounds. Yet, undesirable effects of lanthanum have been observed with other REE forms [31,63,64], primarily with the nitrate form [30] that is more soluble than the oxide and the phosphate forms. Further research is thus needed to unravel the effect of REEs compared to their anions on plant phytotoxicity.

### Uptake and accumulation

All species tested in this experiment were found to uptake and accumulate REEs from soils. Uptake and accumulation of REEs by the roots and shoots, respectively, was generally proportional to the doses tested. Accumulation into the shoots by the three native plant species (the most sensitive species) at the analyzed dose closest to the IC25 values also varied. The artificial soil used in this experiment was approximately pH = 6, representing a moderate value for Canadian soils that are more basic in the western Canada, but more acidic in the eastern Canada. In a previous experiment, it was found that at a lower soil pH (4.08), cerium (Ce) was more toxic to the majority of plant species than at a higher soil pH (6.74), and that Ce uptake by roots and accumulation in shoots was also generally much greater at lower soil pHs [35]. Tyler and Olsson [65] also studied the effects of soil pH on the uptake of metals by a grass, *Agrostis capillaris* L. For the lanthanoids, it was observed that root concentrations were inversely related to soil pH and positively correlated with soil concentration. Other research has indicated that soil pH plays a vital role in the bioavailability of various REEs and their release into soils [65,66,67,68]. It is probable that in a worse-case scenario, where soil has become acidic, REE contamination would become more toxic to plants growing at the site.

As has been found in previous REE studies [27,35,42,65,69,70,71], accumulation of REEs was higher in the roots than in the shoots on a dry biomass basis. Specifically, Tyler and Olsson [28] found slightly lower concentrations of Pr, Nd, Sm, Tb, Dy and Er in the leaves of various species as compared to the roots. Fu et al. [27] observed concentrations ranging from 0.019 to 0.595, 0.001 to 0.097, and 0.0007 to 0.104 mg REE/kg dry weight in the roots, leaves, and stems, respectively, of ferns (*Matteuccia* Todaro spp.) in soils containing approximately 0.191 to 1.697 mg/kg of the six REEs studied in this experiment. Those results, as well as those of Markert and Li [26], were in congruence with our detected plant concentrations at lower doses. Zhang et al. [71] found significantly higher concentrations of REEs in ferns than in other plant groups, as well as higher concentrations in leaves than roots, with root:soil and leaf:soil ratios much greater than those reported in this study (maximums of 7.26 (Sm) for root:soil and 16.07 (Nd) for leaf:soil). Wyttenbach et al. [72] observed Nd, Sm, and Tb leaf concentrations ranging from 0.033 to 0.544 mg/kg, 0.006 to 0.103 mg/kg and ~0.001 to 0.016 respectively in a variety of species grown in soils containing approximately 15.00 mg Nd/kg, 2.82 mg Sm/kg and 0.381 mg Tb/kg. Shoot to soil ratios varied between 0.002 (*Rubus fruticosus* L. for Sm and Tb) to 0.044 (*Acer pseudoplantus* L. for Tb) which is in concordance with the ratios observed for lower soil concentrations in our study (0.006 to 0.050). Another comprehensive experiment comparing 36 plant species grown in natural soils in Japan only detected REEs in 10 species, and only detected our six study REEs in up to three species: *Dicranopteris dichotoma* (Thunb.) Bernh., *Athyrium yokoscense* (Fr. & Sav.) C. Ch. and *Phytolacca americana* L.; however, detected soil concentrations were also low [44]. It was found that for *Phytolacca americana*, a species also native to Canada, leaf concentrations were sometimes higher than the corresponding soil concentrations [44]. On the high end, França et al. [29] measured Nd, Sm, and Tb leaf concentrations of tropical plants grown in Brazilian soils and detected REE concentrations ranging between 18 to 36 mg Nd/kg, 1.9 to 4.9 mg Sm/kg, and 0.24 to 0.47 mg Tb/kg to be < 1.5–28 mg Nd/kg, 0.019–4.2 mg Sm/kg, and < 0.009–0.24 mg Tb/kg respectively. As with several other studies, Pr, Dy and Er were not measured. Accumulation of REEs by plants at the lower doses in these experiments appears to correspond to rates reported for similar, natural REE soil concentrations. Unfortunately, information for higher soil concentrations is lacking. In all cases it can be seen that accumulation rates vary with plant group as well as with species; therefore, testing a variety of plants other than the commonly used crops is highly desirable.

The REE plant:soil ratios, also referred to as transfer factors, in the present study were found to range from 0.001 to 0.278 for aboveground parts with only five of the 120 values above 0.100, with no immediate increase (hyper-accumulation) or decrease (plateauing) in absorption relative to the increasing soil concentrations. In contrast, the transfer factors of the aboveground system varied between 0.010 to 0.369 with 63 of the 120 values at or above 0.100. This is higher than values reported in other studies [26,41,73]; however, our control results were on par with both the leaf:soil ratios (average = 0.051 to 0.276 for Pr, Nd and Sm, respectively) and root:shoot ratios (average = 0.105 to 0.442) obtained where only background soil concentrations were considered [74].

There is no indication in the literature that REEs are essential to plants [20]. Some studies suggest that they may replace calcium and hence cause growth stimulation at low doses [66], although this has been disputed [30,31]. In China, Changle and Nongle, two commercial formulations comprising the nitrate form of REEs are used as seed treatment or sprayed at low doses on crops as fertilizers with demonstrated positive effects ([75] and references therein). Other studies conducted in hydroponic cultures have also demonstrated some growth stimulations. This was not found in the present study in plants grown in low dose contaminated soils. Conversely, higher soil levels of REEs that may arise in the vicinities of mining areas, landfills or where phosphate fertilizers are recurrently applied may be a concern for native terrestrial primary producers. The present study indicated that reductions in both aboveground and belowground biomass of wild native plant species did occur in the presence of elevated soil levels of REEs, therefore monitoring of sites near REE mines and processing facilities is of great importance.

## Supporting Information

S1 Fig Effects of chloride on the plant species tested in the rare earth element (REE) experiments. Calcium chloride was used at doses relevant to those tested with the rare earth elements. Two control doses (one with calcium carbonate, CaCO_3_, added to balance the Ca level with the highest Cl dose, and one with no CaCO_3_ added) were used to examine the effect of calcium alone on plant biomass. Letters above bars represent results of Tukey’s honestly-significant-difference post hoc test. Different letters represent significant differences.
(TIFF)

S1 Table Average percent germination and average speed of germination (both ± standard error) per pot of the five tested plant species grown in increasing soil concentrations (nominal doses 1 to 7) of six rare earth elements (REEs). (XLSX)

S2 Table Analyses (ANOVA or Kruskal-Wallis test) of the effects of rare earth element (REE) soil dosage on the percent germination of five plant species. Post hoc tests [Dunnett's one-sided (ANOVA) or Conover-Inman (Kruskal-Wallis)] were performed in order to determine doses that negatively differed from the controls.
(XLSX)

S3 Table Summary of ANOVA or Kruskal-Wallis analyses on aboveground, shoot biomass (and including the radish bulb for *R*. *sativus*) for five plant species grown in rare earth element (REE) contaminated soils. No observed effect (NOEC) and lowest observed effect (LOEC) concentrations of the given REE were determined based on either Dunnett's one-way post hoc comparisons (ANOVA) or Conover-Inman post hoc comparisons (Kruskal-Wallis) for negative differences (i.e. reduced biomass) from the controls.
(XLSX)

S4 Table ANOVA (or Kruskal-Wallis) results for root biomass for five plant species tested in rare earth element (REE) contaminated soils. Only the control, 2^nd^, 4^th^, and 6^th^ doses for each REE were included. Dunnett's one-way post hoc comparison (or the Conover-Inman test) was used to determine doses that were significantly negatively different from the controls.
(XLSX)

S5 Table Root:soil and shoot:soil ratios based on detected rare earth element (REE) concentrations in dosed soils as well as in the roots and shoots of the five tested plant species. (XLSX)

## References

[1] Hu Z, Haneklaus S, Sparovek G, Schnug E. Rare earth elements in soils. Commun Soil Sci Plant Anal. 2006; 37: 1381–1420.

[2] Stone R. As China’s rare earth R&D becomes even more rarefied, others tremble. Science. 2009; 325: 1336–1337. 10.1126/science.325_1336 19745130

[3] Rüttinger L, Feil M. Sustainable prevention of resource conflicts: new risks from raw materials for the future? Case study and scenarios for China and rare earths. Section Report 3.4, Research Project FKZ 370819 102. adelphi, Berlin, Germany. 2010 [cited 2015 Jan 12]. Available: http://www.adelphi.de/files/de/news/application/pdf/rohkon_report_3.4_china.pdf.

[4] Hurst C. China’s rare earth elements industry: What can the West learn? Institute for the Analysis of Global Security (IAGS). U.S. Army Foreign Military Studies Office, Fort Leavenworth, KS. 2010 [cited 2015 Jan 12]. Available: http://www.iags.org/rareearth0310hurst.pdf.

[5] Avalon Rare Metals Inc. Nechalacho rare earth elements project. 2015 Feb 10 [cited 2015 Apr 22]. Available: http://www.avalonraremetals.com/_resources/factsheet/ProjectSheet.pdf.

[6] Quest Rare Minerals Ltd. Strange Lake rare earth project. 2014 [cited 2015 Jan 12]. Available: http://www.questrareminerals.com/strange_lake.php.

[7] Long KR, Van Gosen BS, Foley NK, Cordier D. The principle rare earth element deposits of the United States: a summary of domestic deposits and a global perspective In: Sinding-Larsen R, Wellmer F-W, editors. Non-Renewable Resource Issues: Geoscientific and Societal Challenges. Netherlands: Springer; 2012 p. 131–155. 10.1007/978-90-481-8679-2_7

[8] United States Department of Energy. Critical Materials Strategy. 2010 Dec [cited 2015 Jan 12]. Available: http://energy.gov/node/206101.

[9] Du X, Graedel TE. Global rare earth in-use stocks in NdFeB permanent magnets. J Ind Ecol. 2011; 15(6): 836–843.

[10] de Boer MA, Lammertsma K. Scarcity of rare earth elements. Chem Sus Chem. 2013; 6: 2045–2055. 10.1002/cssc.201200794 24009098

[11] Hayes-Labruto L, Schillebeeckx SJD, Workman M, Shah N. Contrasting perspectives on China’s rare earth policies: reframing the debate through a stakeholder lens. Energy Policy. 2013; 63: 55–68.

[12] Gonzalez V, Vignati DAL, Leyval C, Giamberini L. Environmental fate and ecotoxicity of lanthanides: Are they a uniform group beyond chemistry? Environ Intern. 2014; 71: 148–157. 10.1016/j.envint.2014.06.019 25036616

[13] Volokh AA, Gorbunov AV, Gundorina SF, Revich BA, Frontasyeva MV, Pal CS. Phosphorus fertilizer production as a source of rare-earth elements pollution of the environment. Sci Total Environ. 1990; 95: 141–148. 2169646 10.1016/0048-9697(90)90059-4

[14] Turra C, Fernandes EAN, Bacchi MA. Evaluation on rare earth elements of Brazilian agricultural supplies. J Environ Chem Ecotoxicol. 2011; 3(4): 86–92.

[15] Yang Q, Wang L, Zhou Q, Huang X. Toxic effects of heavy metal terbium ion on the composition and functions of cell membrane in horseradish roots. Ecotox Environ Saf. 2015; 111: 48–58. 10.1016/j.ecoenv.2014.10.002 25450914

[16] IPNI (International Plant Nutrition Institute). World production of phosphate rock. Better Crops. 1999 [cited 2015 Jan 12]; 83(1): 4–7. Available: http://www.ipni.net/publication/bettercrops.nsf/0/226AFC1599C06D538525798000820184/$FILE/Better%20Crops%201999-1%20p04.pdf.

[17] Sneller FEC, Kalf DF, Weltje L, Van Wezel AP. Maximum permissible concentrations and negligible concentrations for rare earth elements (REEs). RIVM Report 601501 011. National Institute of Public Health and the Environment, the Netherlands. 2000 [cited 2015 Jan 12] Available: http://rivm.openrepository.com/rivm/bitstream/10029/9551/1/601501011.pdf.

[18] Slooff W, Bont PFH, van den Hoop MAGT, Janus JA, Annema JA. Exploratory report: rare earth metals and their compounds. Bilthoven, the Netherlands: National Institute of Public Health and Environmental Protection; 1993. Report No. 710401025.

[19] Li J, Hong M, Yin X, Liu J. Effects of the accumulation of the rare earth elements on soil macrofauna community. J Rare Earth. 2010; 28(6): 957–964.

[20] Liang T, Li K, Wang L. State of rare earth elements in different environmental components in mining areas of China. Environ Monit Assess. 2014; 186:1499–1513 10.1007/s10661-013-3469-8 24135922

[21] Pickard BG. Comparison of calcium and lanthanum ions in the *Avena*-coleoptile growth test. Planta. 1970; 91: 314–320. 10.1007/BF00387504 24500095

[22] Hu Z, Richter H, Sparovek G, Schnug E. Physiological and biochemical effects of rare earth elements on plants and their agricultural significance: a review. J Plant Nutr. 2004; 27(1): 183–220.

[23] Liu M, Hasenstein KH. La^3+^ uptake and its effect on the cytoskeleton in root protoplasts of Zea mays L. Planta. 2005; 220: 658–666. 15449062 10.1007/s00425-004-1379-2

[24] Babula P, Adam V, Opatrilova R, Zehnalek J, Havel L, Kizek R. Uncommon heavy metals, metalloids and their plant toxicity: a review. Environ Chem Lett. 2008; 6: 189–213.

[25] Xiaoqing L, Hao H, Chao L, Min Z, Fashui H. Physico-chemical property of rare earths—effects on the energy regulation of photosystem II in Arabidopsis thaliana. Biol Trace Elem Res. 2009; 130: 141–151. 10.1007/s12011-009-8321-1 19221699

[26] Markert B, Li ZD. Natural background concentrations of rare-earth elements in a forest ecosystem. Sci Total Environ. 1991; 103: 27–35.

[27] Fu F, Akagi T, Shinotsuka K. Distribution pattern of rare earth elements in fern: implication for intake of fresh silicate particles by plants. Biol Trace Elem Res. 1998; 64: 13–26. 9845459 10.1007/BF02783321

[28] Tyler G, Olsson T. Rare earth elements in forest-floor herbs as related to soil conditions and mineral nutrition. Biol Trace Elem Res. 2005; 106: 177–191. 16116249 10.1385/BTER:106:2:177

[29] França EJ, De Nadai Fernandes EA, Turra C, Bacchi MA, Elias C, Tagliaferro F, et al Survey of lanthanoids in plants from a tropical region. Int J Environ Heal. 2011; 5(1–2): 32–48.

[30] Hu X, Ding Z, Chen Y, Wang X Dai L. Bioaccumulation of lanthanum and cerium and their effects on the growth of wheat (*Triticum aestivum* L.) seedlings. Chemosphere. 2002; 48: 621–629. 12143937 10.1016/s0045-6535(02)00109-1

[31] Diatloff E, Smith FW, Asher CJ. Effects of lanthanum and cerium on the growth and mineral nutrition of corn and mungbean. Ann Bot. 2008; 101: 971–982. 10.1093/aob/mcn021 18292604 PMC2710236

[32] Zhang S, Shan XQ. Speciation of rare earth elements in soil and accumulation by wheat with rare earth fertilizer application. Environ Pollut. 2001; 112: 395–405. 11291446 10.1016/s0269-7491(00)00143-3

[33] Pang X, Li D, Peng A. Application of rare-earth elements in the agriculture of China and its environmental behavior in soil. Environ Sci Pollut Res Int. 2002; 9(2): 143–148. 12008295 10.1007/BF02987462

[34] Zhang C, Li Q, Zhang M, Zhang N, Li M. Effects of rare earth elements on growth and metabolism of medicinal plants. Acta Pharmaceutica Sinica B. 2013; 3(1): 20–24.

[35] Thomas P, Carpenter D, Boutin C, Allison JE. Rare earth elements (REEs): effects on germination and growth of selected crop and native plant species. Chemosphere. 2014; 96: 57–66. 10.1016/j.chemosphere.2013.07.020 23978671

[36] Kataoka T, Stekelenburg A, Nakanishi TM, Delhaize E, Ryan PR. Several lanthanides activate malate efflux from roots of aluminium-tolerant wheat. Plant Cell Environ. 2002; 25: 453–460.

[37] d'Aquino L, de Pinto MC, Nardi L, Morgana M, Tommasi F. Effect of some light rare earth elements on seed germination, seedling growth and antioxidant metabolism in *Triticum durum* . Chemosphere. 2009; 75(7): 900–905. 10.1016/j.chemosphere.2009.01.026 19215958

[38] Wang L, Zhou Q, Huang X. Photosynthetic responses to heavy metal terbium stress in horseradish leaves. Chemosphere. 2009; 77: 1019–1025. 10.1016/j.chemosphere.2009.07.065 19712958

[39] Cowgill UM. Biogeochemical cycles for the chemical elements in *Nymphaea odorata* Ait. and the aphid *Rhopalosiphum nymphaeae* (L.) living in Linsley Pond. Sci Total Environ. 1973; 2: 259–303.

[40] Greenwood NN, Earnshaw A. Chemistry of the Elements. 2nd ed Great Britain: Butterworth-Heinemann, Reed Educational and Professional Publishing Ltd; 1997.

[41] Tyler G. Rare earth elements in soil and plant systems—a review. Plant Soil. 2004; 267: 191–206.

[42] Brioschi L, Steinmann M, Lucot E, Pierret MC, Stille P, Prunier J, et al Transfer of rare earth elements (REE) from natural soil to plant systems: implications for the environmental availability of anthropogenic REE. Plant Soil. 2012; 366(1–2): 143–136. 10.1016/j.jcis.2011.09.059 22014394

[43] Markert B. The pattern of distribution of lanthanide elements in soils and plants. Phytochemistry. 1987; 26(12): 3167–3170.

[44] Ichihashi H, Morita H, Tatsukawa R. Rare earth elements (REEs) in naturally grown plants in relation to their variation in soils. Environ Pollut. 1992; 76:157–162. 15091997 10.1016/0269-7491(92)90103-h

[45] Patnaik P. Handbook of Inorganic Chemicals. USA: McGraw-Hill; 2003

[46] Yoshida S, Muramatsu Y, Tagami K, Uchida S. Concentrations of lanthanide elements, Th, and U in 77 Japanese surface soils. Environ Int. 1998; 24(3): 275–286.

[47] Masau M, Černý P, Chapman R . Dyprosian xenotime-(Y) from the Annie Claim #3 granitic pegmatite, southeastern Manitoba, Canada: evidence of the tetrad effect? Can Mineral. 2000; 38: 899–905.

[48] Mackenzie RC. A micromethod for determination of cation-exchange capacity of clay. J Colloid Sci. 1951; 6: 219–222.

[49] Oldham MJ, Bakowsky WD, Sutherland DA. Floristic Quality Assessment System for Southern Ontario. Report. Peterborough, Ontario, Canada: Natural Heritage Information Centre, Ontario Ministry of Natural Resources; 1995

[50] United States Environmental Protection Agency (USEPA) (1996). Method 1638—Determination of trace elements in ambient waters by inductively coupled plasma—mass spectrometry. Washington, D.C.: USEPA 1996 [cited 2015 Jan 12]; Available: http://water.epa.gov/scitech/methods/cwa/bioindicators/upload/2007_07_10_methods_method_1638.pdf.

[51] Boutin C, Carpenter D, Thomas P. Lanthanum chloride (LaCl_3_): effects on crops and selected native plant species Report produced for the Inorganics Unit, Ecological Assessment Division, Science and Technology Branch of Environment Canada. Ottawa, Ontario: Environment Canada Ottawa; 2012.

[52] Environment Canada. Biological test method: test for measuring emergence and growth of terrestrial plants exposed to contaminants in soil. Report. Ottawa, Ontario: Method Development and Applications Section, Environmental Technology Centre. 2005 Feb (amended 2007 Jun). Report No.: EPS 1/RM/45—February 2005 (with June 2007 amendments).

[53] Norberg-King TJ. A linear interpolation method for sublethal toxicity: the inhibition concentration (ICp) approach (version 2.0) Technical Report. Duluth, MN: U.S. Environmental Protection Agency, Environmental Research Laboratory 1993. Report No.: 03–93.

[54] Carpenter D, Boutin C, Parsons J, Ellis DM. Heavy rare earth elements terbium, erbium, and dysprosium: effects on selected crops and native plant species Report produced for the Inorganics Unit, Ecological Assessment Division, Science and Technology Branch of Environment Canada. Ottawa, Ontario: Environment Canada Ottawa; 2014.

[55] Carpenter D, Allison JE, Boutin C. Samarium chloride hexahydrate (SmCl_3_·6H_2_O): effects on selected crops and native plant species Report produced for the Inorganics Unit, Ecological Assessment Division, Science and Technology Branch of Environment Canada. Ottawa, Ontario: Environment Canada Ottawa; 2013.

[56] Bar Y, Apelbaum A, Kafkafi U, Goren R. Relationship between chloride and nitrate and its effects on growth and mineral composition of avocado and citrus plants. J Plant Nutr. 1997; 20(6):715–731.

[57] Bañuls J, Primo-Millo E. Effects of chloride and sodium on gas exchange parameters and water relations of *Citrus* plants. Physiol Plant. 1992; 86: 115–123.

[58] Fashui H, Weiping S, Zhigang W, Mingliang Y, Jia Y, Jiajia Y, et al Effect of La(III) on the growth and aging of root of loquat plantlet in vitro. Biol Trace Elem Res. 2005; 104(2): 185–191. 15894818 10.1385/BTER:104:2:185

[59] Shi P, Huang Z, Chen G. Influence of lanthanum on the accumulation of trace elements in chloroplasts of cucumber seedling leaves. Biol Trace Elem Res. 2006; 109:181–188. 16444007 10.1385/BTER:109:2:181

[60] Xie ZB, Zhu JG, Chu HY, Zhang YL, Zeng Q, Ma HL, et al Effect of lanthanum on rice production, nutrient uptake, and distribution. J Plant Nutr. 2002; 25(10): 2315–2331.

[61] Zeng FL, Zhang MF, Zhou SM, Wu JG, Deng RW. The effect of lanthanide chloride on abscisic acid and electron-transport activity of some crops. Biol Trace Elem Res. 1999; 67: 277–284. 10201334 10.1007/BF02784427

[62] Zeng Q, Zhu JG, Cheng HL, Xie ZB, Chu HY. Phytotoxicity of lanthanum in rice in haplic acrisols and cambisols. Ecotox Environ Saf. 2006; 64: 226–233. 16406581 10.1016/j.ecoenv.2005.03.016

[63] Fashui H, Ling W, Chao L. Study of lanthanum on seed germination and growth of rice. Biol Trace Elem Res. 2003, 94:273–286. 12972694 10.1385/BTER:94:3:273

[64] He YW, Loh CS. Cerium and lanthanum promote floral initiation and reproductive growth of *Arabidopsis thaliana* . Plant Sci. 2000; 159:117–124. 11011099 10.1016/s0168-9452(00)00338-1

[65] Tyler G, Olsson T. Plant uptake of major and minor mineral elements as influenced by soil acidity and liming. Plant Soil. 2001; 230: 307–321.

[66] Cao X, Chen Y, Wang X, Deng X. Effects of redox potential and pH value on the release of rare earth elements from soil. Chemosphere. 2001; 44: 655–661. 11482653 10.1016/s0045-6535(00)00492-6

[67] Yufeng Z, Zhenghua W, Xiaorong W, Lemei D, Yijun C. Mobility of the rare earth elements with acid rainwater leaching in the soil column. Bull Environ Contam Toxicol. 2001; 67: 399–407. 11479670 10.1007/s001280138

[68] von Tucher S, Schmidhalter U. Lanthanum uptake from soil and nutrient solution and its effects on plant growth. J Plant Nutr Soil Sci. 2005; 168: 574–580.

[69] Fu F, Akagi T, Yabuki S Iwaki M. The variation of REE (rare earth elements) patterns in soil-grown plants: a new proxy for the source of rare earth elements and silicon in plants. Plant Soil. 2001; 235: 53–64.

[70] Zhang S, Shan XQ. Speciation of rare earth elements in soil and accumulation by wheat with rare earth fertilizer application. Environ Pollut. 2001; 112: 395–405. 11291446 10.1016/s0269-7491(00)00143-3

[71] Zhang ZY, Wang YQ, Li FL, Xiao HQ, Chai ZF. Distribution characteristics of rare earth elements in plants from a rare earth ore area. J Radioanal Nucl Chem. 2002; 252:461–465.

[72] Wyttenbach A, Furrer V, Schleppi P, Tobler L. Rare earth elements in soil and in soil-grown plants. Plant Soil. 1998; 199: 267–273.

[73] Bibak A, Stürup S, Knudsen L, Gundersen V. Concentrations of 63 elements in cabbage and sprouts in Denmark. Commun Soil Sci Plant. 1999; 30, 2409–2418.

[74] Wahid PA, Kamalam NV, Prabhu RK, Sekhar JK, Vijayalakshmi S, Mahalingam TR, et al Rare earth element fluxes in diverse soils and their absorption by coconut palm. J Plant Nutr. 2003; 26(7): 1427–1438.

[75] Redling K (2006) Rare earth elements in agriculture with emphasis on animal husbandry [PhD dissertation]. München: Ludwig-Maximilians-Universität. 2006 [cited 2015 Jan 12]. Available: http://edoc.ub.uni-muenchen.de/5936/.

