# Supplementary figures and images for: Uptake and Effects of Six Rare Earth Elements (REEs) on Selected Native and Crop Species Growing in Contaminated Soils
Source: PLoS One. 2015 Jun 15;10(6):e0129936. doi: 10.1371/journal.pone.0129936 (PMC4468158; doi:10.1371/journal.pone.0129936)

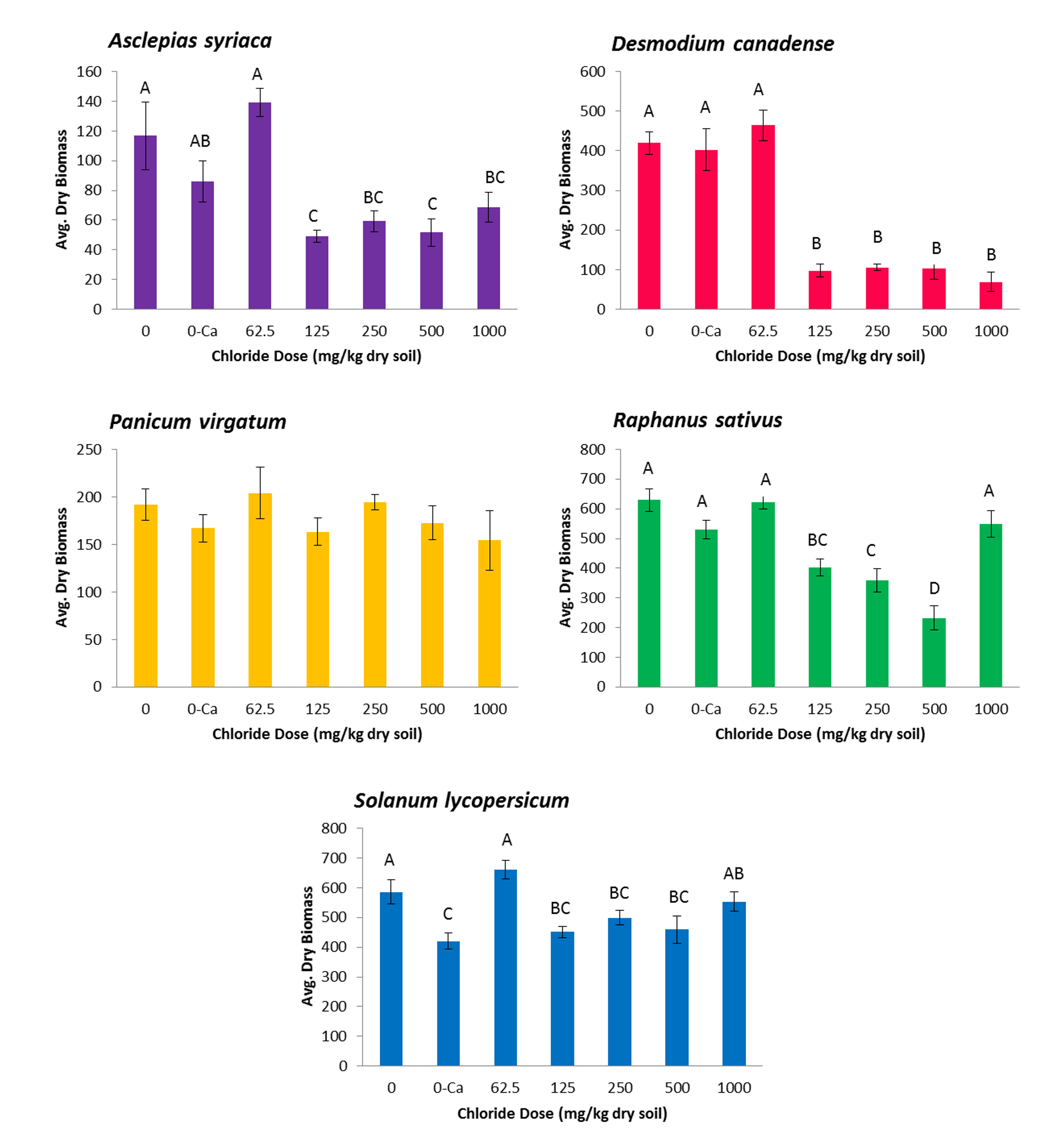

Supplement: S1 Fig — Calcium chloride was used at doses relevant to those tested with the rare earth elements. Two control doses (one with calcium carbonate, CaCO3, added to balance the Ca level with the highest Cl dose, and one with no CaCO3 added) were used to examine the effect of calcium alone on plant biomass. Letters above bars represent results of Tukey’s honestly-significant-difference post hoc test. Different letters represent significant differences. (TIFF) [file pone.0129936.s001.tiff]
